# Diet in Diabetes

**Published:** 1864-05

**Authors:** 


### DIET IN DIABETES.

Dr. Edward Smith concludes some interesting observations on this subject with the following summary of the proper diet in diabetes:

1. *Fluids.*—To be limited by degrees daily until they shall not exceed five pounds and a half in both fluid and solid food. Of this quantity two to three pints should consist of new or skimmed milk, and one pint, or less, of tea. In the cold season and at night they should always be given when hot. Of all alcohols brandy is the best, and may be given with water only, or added to milk, or beat up with egg and milk, and given several times daily. No fluid should be given in greater quantity than half a pint at a time, and when milk is reduced in volume by cooking, the daily quantity of fluid must be made up by an additional supply of the same or other fluid.

2. *Solids.*—Dr. Prout's combination of eggs and milk (with sharps substituted for bran) is excellent. Four ounces of sharps and 4 oz. of peas, beans, or lentils may be made into bread or pudding, with milk, or into omelets with eggs and herbs. Eggs and gelatin may be given when starchy food cannot be altogether intermitted. Eggs, gelatin, cheese, gluten, bread, meat, fat, and oils may be given as largely as they can be digested. The free use of salad oil should be urged, whether in the cooking of fish or flesh, or in the use of water-cress as a salad, or drunk alone, so that several ounces may, if possible, be consumed daily; but as there are in all persons preferences and dislikes in reference to particular fats, that kind—whether butter, suet, oil, or fat of meat—should be allowed which is the most agreeable. Four oz. of sharps, 3 oz. of wheaten flour, 5 oz. of peas, 1 lb. of meat, 2 oz. of cheese, 2 pints of milk, and 3 eggs, will afford more than about 13 oz. of carbon and 1 oz. of nitrogen daily. —*Lancet, Feb.* 6, 1864.

